# The impact of modifiable risk factor reduction on future dementia burden: a microsimulation modeling study

**DOI:** 10.1007/s10654-025-01283-0

**Published:** 2025-08-23

**Authors:** Chiara C. Brück, Koen de Nijs, M. Arfan Ikram, Frank J. Wolters, Inge M. C. M. de Kok

**Affiliations:** 1 Department of Public Health, Erasmus MC University Medical Center, Rotterdam, The Netherlands; 2 Department of Epidemiology, Erasmus MC University Medical Center, Rotterdam, The Netherlands; 3 Department of Radiology & Nuclear Medicine and Alzheimer Center, Erasmus MC University Medical Center, Rotterdam, The Netherlands

**Keywords:** Dementia, Risk factors, Prevention, Modeling

## Abstract

Approximately 30%-50% of dementia cases are attributable to modifiable risk factors, but the impact of risk reduction strategies on dementia incidence at a population level is uncertain. Reliable estimates of intervention effects require accounting for changes in life expectancy when intervening on risk factors, and model realistic reduction scenarios that consider co-occurrence of risk factors. Using the microsimulation model MISCAN-Dementia, we assessed the effect of interventions on mid-life hypertension and late-life smoking on dementia and mortality risk. We modeled risk factor reductions, from small reductions to complete elimination, and evaluated effects on dementia incidence and prevalence, number of cases, and life years with and without dementia. All risk factor reductions resulted in lower dementia incidence and prevalence, fewer dementia cases, and more dementia-free life years. Eliminating smoking resulted in 1.4% fewer dementia cases for women and 2.5% for men over their lifetime. Eliminating hypertension reduced dementia cases by 1.1% for women and 3.3% for men. The number of dementia cases and life years with dementia decreased until around age 90, after which a slight increase was observed due to prolonged life expectancy with the reductions. Reducing smoking and hypertension will result in additional life years without dementia and a modest reduction in overall dementia cases, with some additional dementia cases in the oldest old. These findings emphasize the potential of dementia risk reduction strategies and the importance of considering concurrent changes in mortality when evaluating risk factor reductions.

**Supplementary Information:**

The online version contains supplementary material available at 10.1007/s10654-025-01283-0.

## Introduction

Dementia presents a significant public health issue and substantial economic burden on health-care systems and society [1]. Worldwide, more than 55 million people are living with dementia and due to population ageing this number is expected to increase in the coming decades [2]. Risk reduction strategies are urgently needed to curb the dementia burden. Up to 45% of all dementia cases are attributable to 14 modifiable risk factors [3]. These factors, which increase dementia risk, can arise in early life (e.g. education), mid-life (e.g. obesity, traumatic brain injury, hypertension) and late-life (e.g. smoking, depression, social isolation) [3, 4].

These modifiable risk factors present an important opportunity for clinical and public health interventions for the prevention of dementia. However, the effect of public health interventions targeting risk factors is still unknown. A reduction or elimination of a risk factor does not necessarily translate into an equal reduction in dementia incidence or prevalence, because the reduction of a risk factor often also prolongs life expectancy. As the incidence of dementia rises exponentially with age [5], the beneficial effect of risk factor reductions on dementia risk might be partially offset by individuals living longer and developing dementia at a later age. Moreover, the causal relationship between risk factors and dementia has not been conclusively established. It likely involves indirect mechanisms and complex, cascading interactions; adding to the challenge of translating reductions in risk factors to reductions in dementia risk. Additionally, it is still unclear whether the reduction of a risk factor eliminates its causal influence entirely or merely postpones the onset of dementia to a later stage in life.

Many studies, including the widely cited Lancet Commission report [3], estimated that reducing modifiable risk factors might prevent up to 30−50% of dementia cases [3, 4, 6–10]. These estimates are based on the prevalence of risk factors and the associated increase in dementia risk. However, they do not account for the fact that many risk factors also contribute to increased all-cause mortality, or that individuals frequently present with multiple co-occurring risk factors. These omissions can lead to an overestimation of the potential impact of risk reduction. Accurately estimating the effect of reducing risk factors should also consider their influence on mortality, particularly given that dementia risk increases dramatically with age. Mathematical models that simulate the entire lifetime of individuals are necessary to untangle the relationship between incidence of dementia, reductions in modifiable risk factors and increases in life expectancy.

Observational studies have produced mixed results linking modifiable risk factors with dementia risk. Limitations such as small sample sizes and short follow-up periods hinder the ability to draw firm conclusions. Nevertheless, despite some inconsistencies, there is strong evidence for mid-life hypertension [3, 4, 11–17] and to a lesser extent for late-life smoking [3, 4, 7, 15–23] to increase the risk of cognitive decline and dementia. In addition, both risk factors have clear interventions to mitigate them (such as smoking cessation and hypertension management), and public health initiatives can promote these interventions. Given significant differences in background mortality [24], overall dementia incidence [25], and prevalence of risk factors between men and women [26, 27], providing sex-stratified results offers important insights into the effects of interventions. Therefore, the aim of this study is to investigate the effect of changes in smoking and hypertension prevalence on future dementia outcomes at a population level, accounting for effects of the risk factors on both dementia and mortality risk.

## Methods

To estimate the effect of changes in modifiable risk factors on the future dementia burden, we conducted an analysis using the MISCAN-Dementia microsimulation model [28]. MISCAN-Dementia is a microsimulation model based on the validated Microsimulation Screening Analysis (MISCAN) model [29]. Details of the model development and calibration have been described previously [28]. In brief, MISCAN-Dementia, coded in Python 3.10, is a stochastic microsimulation model that predicts how dementia incidence and prevalence will develop in a population. The model allows for unidirectional stage transitions between cognitively normal, mild cognitive impairment (MCI), mild dementia, moderate dementia, severe dementia, and death due to dementia. In any stage, an individual may also die of other causes.

### Population data

Age- and sex-specific dementia data were derived from participants of the Rotterdam Study, an ongoing population-based cohort study of 17,931 individuals aged > 40 years who reside in the Ommoord suburb of Rotterdam, the Netherlands. Details of the study design and of case ascertainment for dementia and mortality have been described previously [30, 31]. For the current study, we included all 10,209 participants who were alive and non-demented before the start of the fourth examination cycle on 1 January 2002. Information on vital status was obtained regularly from the municipal health authorities in Rotterdam. Participants were screened for dementia at each four-yearly center visit using the Mini-Mental State Examination and the Geriatric Mental Schedule organic level, and screen-positives underwent further investigation and informant interviews, including the Cambridge Examination for Mental Disorders of the Elderly. All participants also underwent routine cognitive assessment, including a verbal fluency test (animal categories), 15-word learning test, letter-digit substitution task, Stroop test, and Purdue pegboard task. In addition, they were under continuous surveillance for the detection of interval cases between center visits, through electronic linkage of the study base with medical records from general practitioners and the regional institute for outpatient mental healthcare. A consensus panel headed by a consultant neurologist established the final diagnosis in accordance with standard criteria for dementia (DSM-III-R). As such, 1,118 of 10,209 participants were diagnosed with dementia during follow-up until 1 January 2016.

In this study, we simulated the life histories of the 1980 Dutch birth cohort, using sex-specific life tables based on demographic data from Statistics Netherlands [24]. Furthermore, the life tables were adjusted for dementia-related mortality [32]. Dementia prevalence and overall life expectancy in the Netherlands lie in the middle of the European distribution [33, 34]. Compared to the United States, the Netherlands has slightly lower age-standardized dementia prevalence [35] and slightly higher life expectancy [34].

### Risk factor definition

This analysis focuses on mid-life hypertension and late-life smoking as modifiable risk factors for dementia. Mid-life hypertension is defined as hypertension between the ages 40 and 59, and late-life smoking as being a current smoker after the age of 65. Prevalence data for hypertension and smoking was taken from Statistics Netherlands [26, 27]. The resulting prevalences are comparable to those of other Western European countries [36, 37]. Mid-life hypertension was adjusted for medication use [38], to best reflect unmedicated hypertension. Of note, fewer than one in seven people with hypertension in this age group use blood pressure lowering medication (Table 1). The co-occurrence of the risk factors was estimated based on data from the Rotterdam Study [31], where 24% of female and 31% of male late-life smokers also had mid-life hypertension. Risk-factor mortality and dementia risks were taken from systematic reviews for the respective age groups [4, 39, 40]. We assumed the effect of both risk factors to be additive. Table 1 summarizes the risk factor parameters.

**Table 1:** Risk factor parameters and sources

Parameter	Female	Male	Source
Prevalence (%, 95% CI)			
Late-life smoking	10.0 (8.6, 11.7)	13.8 (12.1, 15.6)	Statistics Netherlands [26]
Mid-life hypertension	10.0 (9.8, 10.1)	9.3 (9.2, 9.5)	Statistics Netherlands [27]
Hypertension medication	1.7	1.1	Statistics Netherlands [38]
Both	2.4 (2.1, 2.8)	4.3 (3.8, 4.8)	Rotterdam Study [31]
Mortality risk (RR, 95% CI)			
None	1	1	Reference
Late-life smoking	1.9 (1.7, 2.1)	1.8 (1.6, 2.0)	Systematic review [39]
Mid-life hypertension	1.3 (1.1, 1.7)	1.3 (1.1, 1.7)	Systematic review [40]
Both	Assume additive effect		
Dementia risk (RR, 95% CI)			
None	1	1	Reference
Late-life smoking	1.6 (1.2, 2.2)	1.6 (1.2, 2.2)	Systematic review [4]
Mid-life hypertension	1.6 (1.2, 2.2)	1.6 (1.2, 2.2)	Systematic review [4]
Both	Assume additive effect		

### Risk factor calibration

For this analysis, MISCAN-Dementia was further developed to include mid-life hypertension and late-life smoking as risk factors for dementia. The simulated population was divided into four risk groups: none, smoking, hypertension, and both. To reflect the characteristics of these groups, we calibrated the prevalence, dementia and mortality risk of the risk factor groups as outlined in Table 1. Model calibration refers to the process of adjusting model parameters so that the simulation outputs closely match real-world, observed data (called calibration targets). The prevalence of the risk factors was calibrated for the respective age groups (hypertension 40–59 years, smoking 65–100 years). The prevalences in Table 1 were taken as calibration targets and the sizes of the risk groups were the calibrated parameters. For the dementia incidence, the baseline risk for the “none” risk group was the calibrated parameter, which was then multiplied by the relative risks of the risk groups. The calibration target was the overall dementia incidence equal to observed incidence rates from the Rotterdam Study [31], adjusted for a linearly declining trend as observed in a recent meta-analysis of North American and European cohort studies [28, 41]. For the mortality risk, the mortality rates were decreased for the “none” risk group and increased for the other risk groups. The calibration target was the overall mortality rate equal to the projected mortality rate of the 1980 birth cohort based on data of Statistics Netherlands [24]. To optimally capture sex differences in the risk factor characteristics and mortality, the calibration was performed separately for men and women. A genetic algorithm (DEAP [42]) was used for all calibrations. More details on the calibration can be found in Supplement A.

### Base case analysis

The reference scenario in the base case analysis simulates the risk factors using the point estimates outlined in Table 1. The risk factor reduction scenarios decrease the prevalence of the risk factors compared to the reference scenario, while maintaining the dementia and mortality risks of the risk factor groups. To study the maximum effect of reductions in smoking and hypertension on the future dementia burden, three extreme scenarios were modeled: complete elimination of (1) both risk factors, (2) hypertension only and (3) smoking only. As complete elimination of a risk factor is often not feasible in the population, we also modeled reductions by 50%, 25% and 10% for both risk factors as well as for each risk factor separately.

### Outcome measures

Each risk factor reduction scenario was run with 100 million simulated individuals born in 1980, to be able to model the effect of present-day interventions in mid-life on future disease burden. The analysis was performed for men and women separately due to their considerable differences in mortality and dementia incidence in general as well as risk factor characteristics. Every risk factor reduction scenario was evaluated in terms of incidence and prevalence, total dementia cases, and total life years with and without dementia. All outcomes were compared to the reference scenario of no reduction. Furthermore, we calculated the difference in dementia cases and life years with and without dementia by age. Since the reduction scenarios influence the size of the population in each age group (i.e. individuals live longer in the reduction scenarios and therefore more often are part of the older age groups), we also calculated the difference in dementia cases, life years with and without dementia relative to 100,000 life years lived by age.

### Sensitivity analyses

In univariate sensitivity analyses, we varied several uncertain parameters to investigate their influence on the future dementia burden. We re-calibrated the model using the bounds of the 95% confidence intervals of the risk factor prevalence, excess dementia risk, and excess mortality risk of the risk factors (Table 1), resulting in twelve additional models for each sex. The same outcomes for every risk factor reduction scenario were calculated and compared to the main analysis.

In view of average life-expectancies and the larger impact of dementia on loss of quality-adjusted life years before age 85 [43], we performed a sub-group analysis of outcomes until age 85.

## Results

Compared to the reference scenario with contemporary risk factor prevalence, all risk factor reductions resulted in a lower incidence and prevalence of dementia, fewer dementia cases, fewer life years with dementia and more dementia-free life years (Table 2). For all outcome measures, effects were larger for men than for women. Background mortality and smoking prevalence was higher for men than for women. For women, smoking reductions had a slightly larger effect than hypertension reductions for all outcome measures (e.g. prevalence rate: − 3.9% smoking elimination vs. − 3.3% hypertension elimination; Table 2). For men, smoking reductions also had a slightly larger effect than hypertension on incidence and prevalence rates and dementia-free life years, but not on total dementia cases and life years with dementia where hypertension had a larger effect (e.g. dementia cases: − 2.5% smoking elimination vs. − 3.3% hypertension elimination; Table 2). The effect of reducing both risk factors simultaneously had a slightly lower effect than the sum of individual reductions (1−5% lower), except for dementia cases and life years with dementia.

### Potentially avoidable dementia cases

**Fig. 1:**
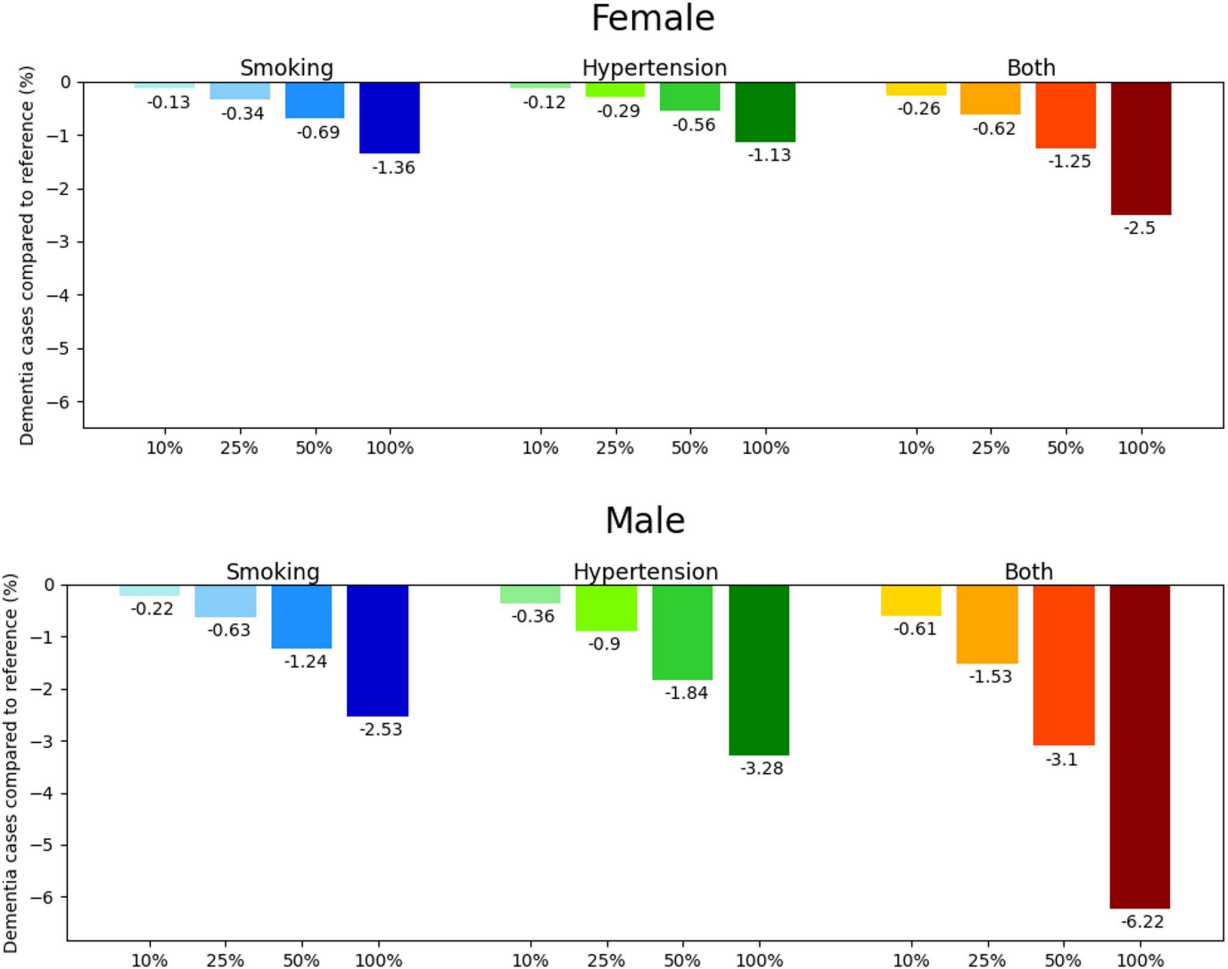
Potentially avoidable dementia cases in percent for all risk factor reduction scenarios compared to the reference scenario

**Fig. 2:**
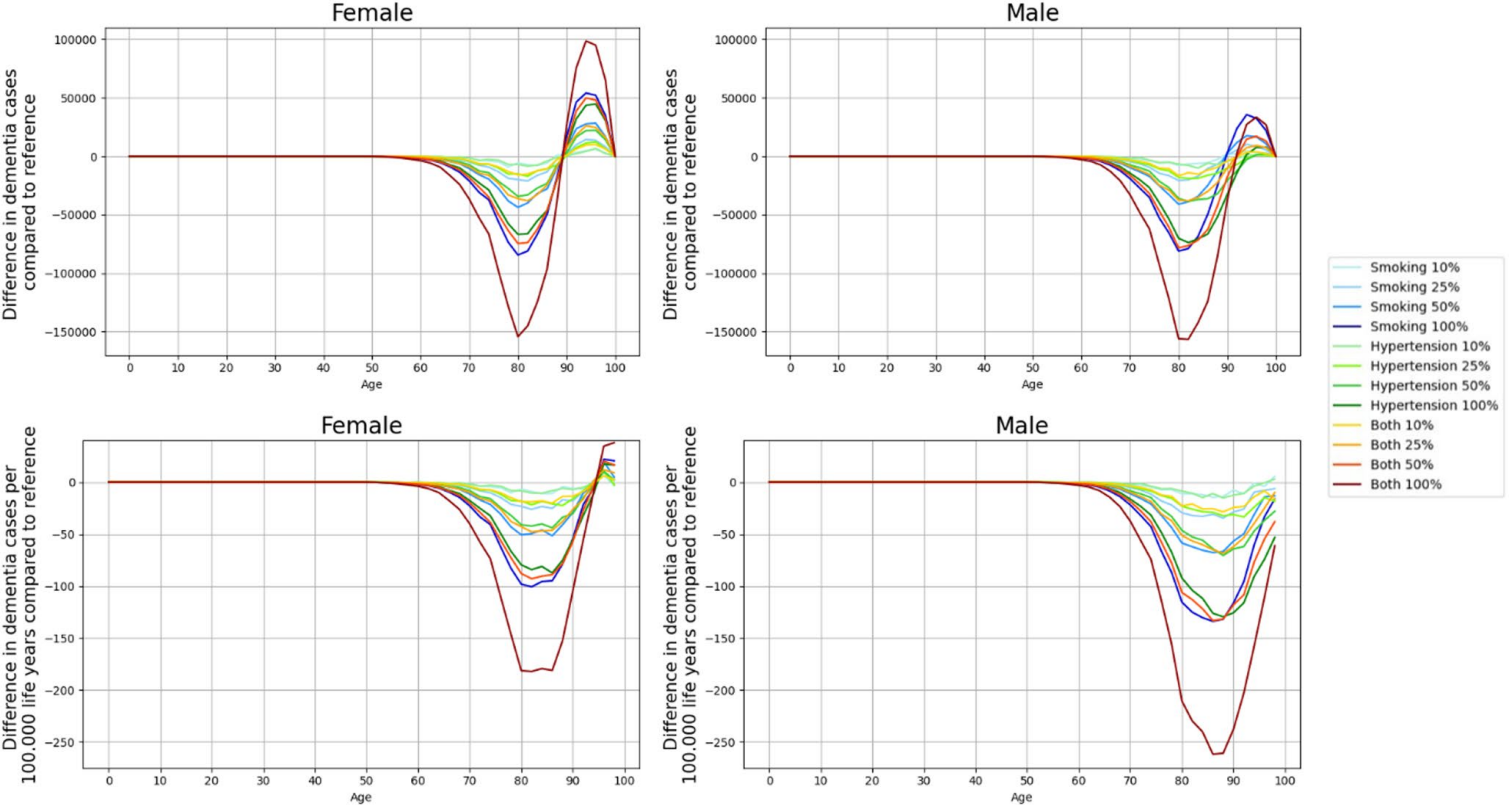
Difference in dementia cases for all risk factor reduction scenarios compared to the reference, top in absolute numbers and bottom relative to 100.000 life years lived by age

Complete elimination of smoking or hypertension led to fewer dementia cases. For women, the reductions were 1.4% for smoking, 1.1% for hypertension, and 2.5% for both. For men, the reductions were 2.5% for smoking, 3.3% for hypertension, and 6.2% for both (Table 2). The effect increases almost linearly with the magnitude of the reduction (Fig. 1). The complete elimination of both risk factors reduced the lifelong probability of developing dementia for an individual free of dementia at age 65 from 54.5 to 53.1% for women and from 35.5 to 33.3% for men (Supplemental Table C3). All reduction scenarios reduced the number of dementia cases in younger ages and then increased for the oldest old (Fig. 2, top panels).The shift from fewer to more dementia cases takes place at age 88 to 90 for women and at age 91 to 96 for men depending on the reduction scenario (Fig. 2, top panels). Whereas we found that absolute cases increased at older ages compared to the reference scenario, the relative rate adjusting for life years lived was reduced for each age and sex group (Fig. 2, bottom panels), except for women over 95 years (Fig. 2, bottom left panel). The complete elimination of both risk factors simultaneously has the largest impact (up to 188 fewer cases for women and 267 cases for men per 100,000 life years; Fig. 2, bottom panels). With a considerable gap, complete elimination of smoking, 50% reduction of both and complete elimination of hypertension have the second to fourth largest effect. To illustrate the size of the population to which these findings apply at various ages, Supplemental tables C1 and C2 provide the corresponding population sizes of all reduction scenarios.

### Life years with and without dementia

**Fig. 3:**
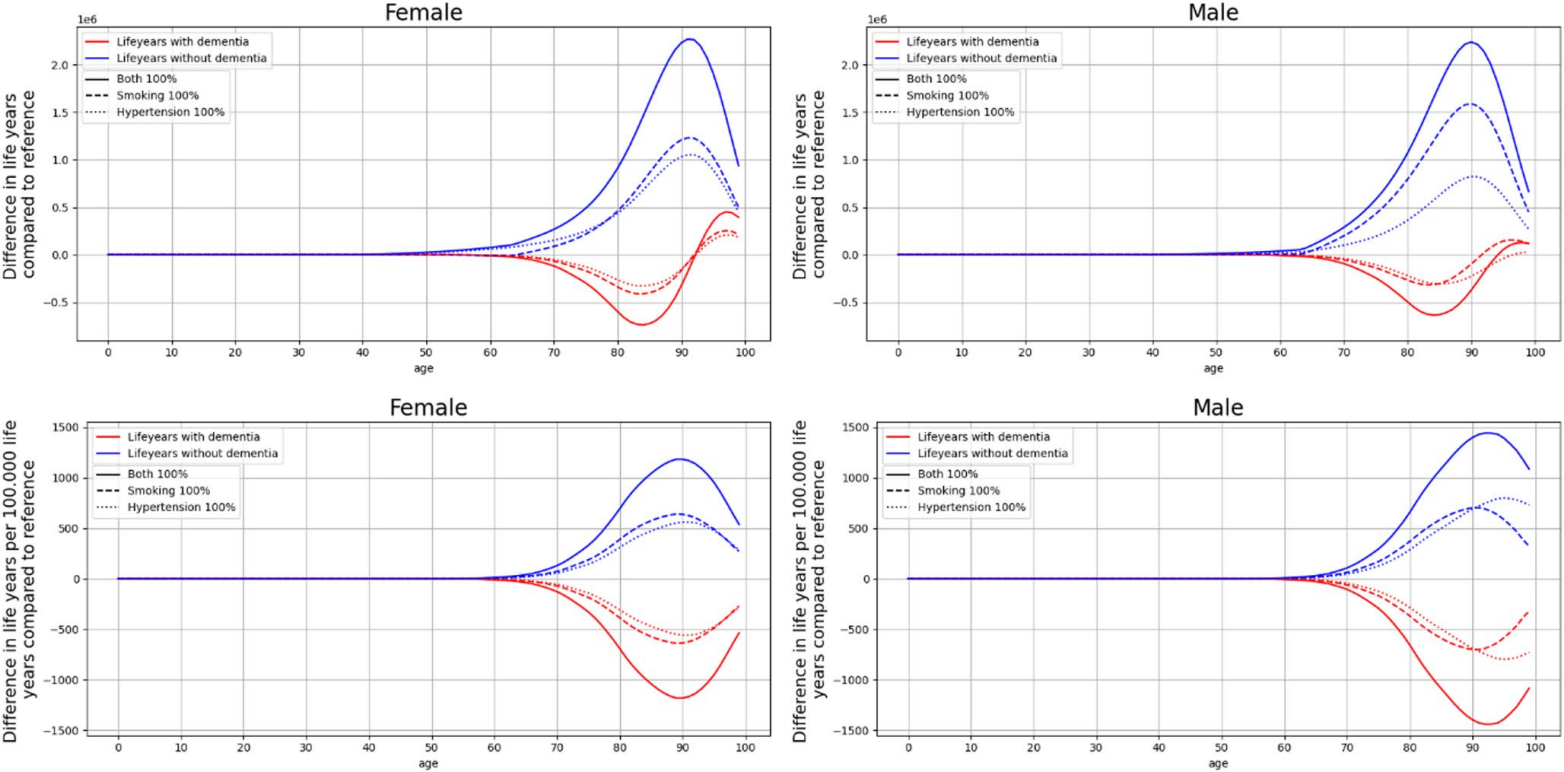
Difference in life years with and without dementia for the three complete elimination scenarios compared to the reference, top in absolute numbers and bottom relative to 100.000 life years lived by age

All reduction scenarios increase the number of life years without dementia in both absolute terms (blue lines in Fig. 3, top panels) and relative to 100,000 life years lived in each age group (blue lines in Fig. 3, bottom panels). The peak in additional life years without dementia is reached around age 90 for both sexes. Similar to reductions in dementia cases, the shift from fewer to more life years with dementia takes place at ages 92 to 94 for women and at ages 92 to 98 for men depending on the reduction scenarios (red lines in Fig. 3, top panels). When considering the life years lived in each age group, there are fewer life years with dementia for both sexes across all ages (red lines in Fig. 3, bottom panels).

**Table 2:** Dementia outcomes by risk factor scenario compared to reference scenario

			Age adjusted incidence rate	Age adjusted prevalence	Total dementia cases, in million	Life years with dementia, in million	Life years without dementia, in million
Female		Ref.	2.22	10.50	51.6	244	8432
	Smoking	10%	2.21 (− 0.37%)	10.46 (− 0.38%)	51.5 (− 0.13%)	243 (− 0.17%)	8434 (0.03%)
		25%	2.20 (− 0.85%)	10.40 (− 0.96%)	51.4 (− 0.34%)	242 (− 0.43%)	8438 (0.07%)
		50%	2.18 (− 1.66%)	10.29 (− 1.98%)	51.2 (− 0.69%)	241 (− 0.91%)	8444 (0.15%)
		100%	2.15 (− 3.21%)	10.09 (− 3.91%)	50.9 (− 1.36%)	239 (− 1.81%)	8457 (0.29%)
	Hypertension	10%	2.21 (− 0.35%)	10.46 (− 0.35%)	51.5 (− 0.12%)	243 (− 0.17%)	8434 (0.03%)
		25%	2.20 (− 0.74%)	10.41 (− 0.83%)	51.4 (− 0.29%)	243 (− 0.37%)	8438 (0.07%)
		50%	2.19 (− 1.41%)	10.33 (− 1.66%)	51.3 (− 0.56%)	242 (− 0.74%)	8444 (0.14%)
		100%	2.16 (− 2.77%)	10.15 (− 3.30%)	51.0 (− 1.13%)	240 (− 1.46%)	8455 (0.27%)
	Both	10%	2.21 (− 0.66%)	10.42 (− 0.73%)	51.4 (− 0.26%)	243 (− 0.33%)	8437 (0.06%)
		25%	2.19 (− 1.53%)	10.31 (− 1.82%)	51.3 (− 0.62%)	241 (− 0.82%)	8444 (0.14%)
		50%	2.15 (− 3.00%)	10.12 (− 3.62%)	50.9 (− 1.25%)	239 (− 1.65%)	8456 (0.29%)
		100%	2.09 (− 5.86%)	9.75 (− 7.12%)	50.3 (− 2.50%)	235 (− 3.31%)	8480 (0.58%)
Male		Ref.	1.56	6.31	32.9	133	8275
	Smoking	10%	1.55 (− 0.59%)	6.27 (− 0.56%)	32.8 (− 0.22%)	132 (− 0.24%)	8278 (0.04%)
		25%	1.54 (− 1.41%)	6.22 (− 1.46%)	32.6 (− 0.63%)	132 (− 0.67%)	8282 (0.10%)
		50%	1.52 (− 2.70%)	6.13 (− 2.84%)	32.4 (−1.24%)	131 (−1.28%)	8290 (0.19%)
		100%	1.48 (− 5.29%)	5.95 (− 5.64%)	32.0 (−2.53%)	129 (−2.61%)	8306 (0.38%)
	Hypertension	10%	1.55 (− 0.56%)	6.28 (− 0.54%)	32.7 (−0.36%)	132 (−0.41%)	8276 (0.02%)
		25%	1.54 (−1.27%)	6.23 (− 1.34%)	32.6 (−0.90%)	131 (−1.01%)	8279 (0.05%)
		50%	1.52 (− 2.48%)	6.14 (− 2.67%)	32.3 (−1.84%)	130 (−2.03%)	8283 (0.10%)
		100%	1.49 (− 4.75%)	5.99 (− 5.15%)	31.8 (−3.28%)	128 (−3.56%)	8294 (0.23%)
	Both	10%	1.54 (− 1.09%)	6.24 (− 1.08%)	32.7 (−0.61%)	132 (−0.64%)	8279 (0.06%)
		25%	1.52 (− 2.57%)	6.14 (− 2.74%)	32.4 (−1.53%)	131 (−1.65%)	8287 (0.15%)
		50%	1.48 (− 5.06%)	5.96 (− 5.47%)	31.8 (−3.10%)	128 (−3.34%)	8299 (0.30%)
		100%	1.41 (− 9.90%)	5.63 (− 10.77%)	30.8 (−6.22%)	124 (−6.70%)	8324 (0.59%)

### Sensitivity analyses

**Fig. 4:**
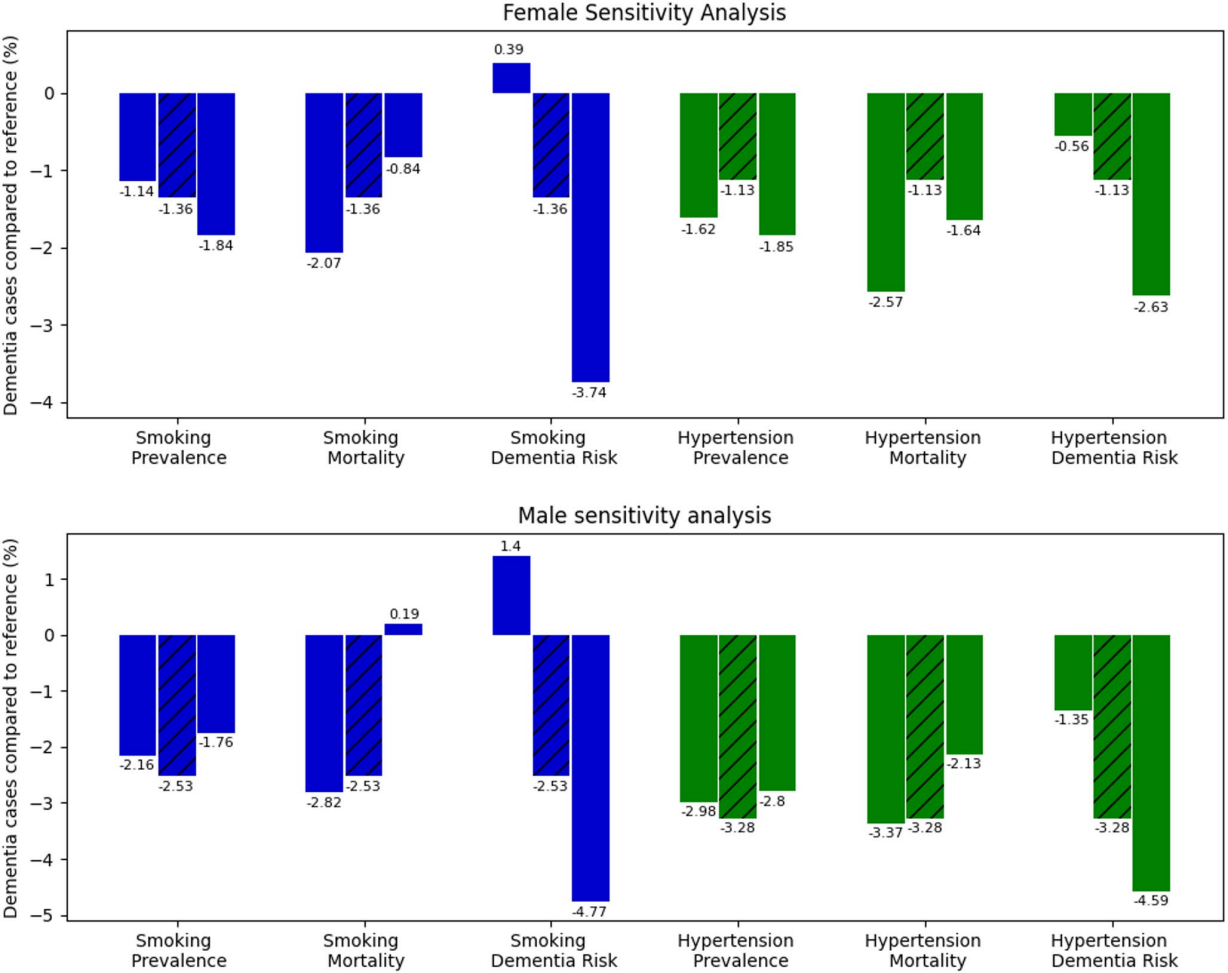
Sensitivity analysis of the risk factor prevalence, mortality and dementia risk on the potentially avoidable dementia cases in percent for the complete smoking (blue) and hypertension (green) compared to the reference scenario

Legend: The main analysis is presented as the middle bar (dashed). The analysis using the lower bound of the parameter is presented as the left bar and the analysis using the upper bound is presented as the right bar. The upper and lower bounds are given in Table 1.

Of all parameters investigated in the sensitivity analysis, the magnitude of the risk factor dementia risk ratios had the largest effect on the results (Fig. 4). The percentage of potentially avoidable dementia cases decreased with a lower dementia risk associated with the risk factors and increased with a higher dementia risk. For hypertension, the percentage remained negative for the entire range of investigated excess dementia risk (1.6 RR, 95% CI 1.2, 2.2, Table 1), meaning also a lower dementia risk associated with hypertension resulted in fewer dementia cases (range − 0.56 to − 2.63% for women and − 1.35 to − 4.59% for men). However, with the lower and upper bounds of the dementia risk associated with smoking (95% CI 1.2–2.2, Table 1), there were more (+) dementia cases compared to the point estimate RR of 1.6 (range *+ *0.39 to − 3.74% for women and *+ *1.40 to − 4.77% for men). Smoking related mortality had the opposite effect; assuming a higher smoking related mortality, reducing smoking prevalence resulted in a lower percentage of potentially avoidable dementia cases. For men, reducing smoking prevalence under the assumption of higher smoking related mortality, even caused an increase (+) in dementia cases compared to the reference (range − 2.82% to *+ *0.19% for men and − 2.07% to − 0.84% for women). All other parameters had a smaller effect on the outcomes.

When restricting the analysis until age 85, the effect of the risk factor reductions becomes more favorable for all outcome measures, except for dementia-free life years (Supplemental Table B1). For example, the beneficial effect of complete risk factor elimination on the number of dementia cases amounts to 7.5% before age 85 for women, as compared to 2.5% across the entire age span (10.6% vs. 6.2% for men). Dementia-free life years are influenced to a lesser extent when excluding the oldest-old (0.58% all ages vs. 0.16% <85 for complete elimination of both risk factors for women).

## Discussion

### Main findings

The reduction of late-life smoking and mid-life hypertension results on a population level in a modestly lower incidence and prevalence of dementia, fewer dementia cases, fewer life years with dementia and more dementia-free life years. These interventions reduce the number of dementia cases and life years with dementia particularly before the age of 90, after which there is a small increase due to increasing life expectancy outweighing dementia incidence reductions. On balance, when considering the life years lived across age groups, reduction scenarios result in fewer dementia cases and life years with dementia.

### Comparison to the literature

Several studies estimated that reducing modifiable risk factors might prevent up to 30–50% of dementia cases [3, 4, 6–10]. These estimates account for risk factor prevalence and dementia risk, but not for risk factor related mortality or the co-occurrence of multiple risk factors. The 2020 Lancet Commission report [4] estimated that the elimination of smoking could potentially prevent 5% of all dementia cases and the elimination of hypertension could potentially prevent 2%. However, the 2024 update [3] revised these estimates to 2% for both smoking and hypertension. This change may reflect the classification of smoking as a mid-life rather than a late-life risk factor in the 2024 report, as well as reduced relative risk estimates for both factors. In the current study, we found similar percentages as the 2024 Lancet Commission report. One prior study from France [44], which also accounted for risk factor mortality when simulating effects of mid-life hypertension elimination, found that the prevalence of dementia reduced by 2%, compared to around 4% in the current study, and reductions in lifetime risk of around 4% compared to around 2% in the current study. Some of the remaining variation between studies may be explained by differences in the modeling strategy and baseline dementia incidence.

### Postponing vs. preventing dementia onset

While narratives around risk factor reduction often claim the prevention of dementia altogether, this framing can be misleading. For a multifactorial disease like dementia, it is important to acknowledge that risk factor interventions likely result in postponement rather than prevention of dementia [45, 46]. Consequently, such interventions may lead to a redistribution of the burden from younger to older age groups, yet still yielding substantial clinical and socioeconomic benefits [46]. Delaying the onset of dementia to older ages alleviates some strain on healthcare systems and caregiving networks, in particular if it leads to so-called “compression of morbidity”. Compression of morbidity refers to a reduction over time in the total lifetime spent with chronic disability [47], occurring when an intervention postpones the onset of disease by more than it increases lifespan. Consequently, in an aging population, life years with and without disease become more meaningful measures than absolute disease risk (i.e. disease incidence). If populations live long enough, eventually most will experience illness. The great success of public health and medicine has been in delaying the onset of illness further and further. It is therefore sensible to examine life years with and without dementia as well as present the analysis for individuals up to age 85. Examining life years with and without dementia shows that interventions shift the burden of dementia to older ages, as illustrated in Figs. 2 and 3 (top panels). Risk factor reductions result in better outcomes for individuals up to age 85 (Supplemental Table B1), since most life years with dementia and mortality gains occur in older age.

### Differences between men and women

For all outcome measures, the effect of risk factor reductions on a population level was more pronounced in men than in women. This disparity is likely due to the higher prevalence of smoking among men, which provides more opportunities for risk factor reduction, and the longer life expectancy in women, which puts them at higher dementia risk in very old age [25]. On an individual and short-term basis, lifestyle interventions showed no significant differences between men and women [48, 49], and some studies reported larger effects for women [50]. These studies, however, featured balanced risk factor exposure between sexes and were generally conducted in age groups with low mortality (between 60 and 80 years). In addition, poor adherence to lifestyle interventions is widespread among men and women, particularly over the long-term [51]. Women tend to have higher adherence to antihypertensive treatments [52]. When translating these effects to the population level and considering risk factor prevalence and background mortality, men benefit more from risk factor reductions than women.

### Timing of primary prevention strategies

Primary prevention interventions targeting risk factors, particularly mid-life risk factors, require a long time horizon since dementia is particularly prevalent in older ages. For the 1980 birth cohort, as simulated in this study, hypertension interventions would take place in the 2020s–2030s and smoking interventions in 2040s, but the effect on dementia would not be fully visible until 2080. Given the long time horizon, the set up and evaluation of primary prevention interventions on a population scale is rarely done [54]. Modeling studies are useful to estimate the long-term effect of primary prevention interventions, and guide transgenerational, long-term policymaking.

### Strengths and limitations

MISCAN-Dementia synthesizes information on the natural history of dementia, birth cohort demographics as well as risk factor characteristics by using input from observational, population-based cohort data and peer-reviewed literature. Although we believe the model provides valid estimates of the effect of risk factor reduction on the future dementia burden, some limitations should be considered. First, the analysis relies on the assumption of causality between modifiable risk factors and the onset of dementia. Whilst such evidence is seldomly available from randomized trials, we believe our analysis represents the current best knowledge on the relationship between the risk factors and dementia and provides valid estimates for guiding preventive strategies. Second, our results are subject to uncertainties in the risk estimation and modeling approach, which we attempted to address by performing sensitivity analyses of the most uncertain model parameters. Risk factor related dementia risk had the biggest impact, and more precise evidence from observational studies may strengthen future modeling approaches. Third, we assumed that reducing a risk factor lowers the dementia risk to the level of non-exposed individuals. Additionally, we did not account for adherence to lifestyle interventions, which is generally low for individual-level interventions - particularly over the long term [51]. While this is a simplification of reality, it illustrates the maximum potential effect of risk factor reductions in a population. Fourth, we could not directly model quality-of-life effects as these depend on many factors beyond dementia outcomes. As a proxy for quality-of-life impact, in the analysis by age and the sub-group analysis focusing on individuals aged 85 or younger, we found that the effect of risk factor reductions was more favorable for younger age groups. Fifth, the model was developed based on Dutch data. Although risk factor and dementia occurrence are fairly comparable to other high-income countries in Europe and North America [36, 37], this limits generalizability of the results to other populations worldwide.

## Conclusions

The reduction of smoking and hypertension can be expected to result in a meaningful reduction of future dementia occurrence. Risk factor reduction lowered the number of dementia cases and life years with dementia particularly until age 90, postponing some of the burden of dementia until later in life. Risk reduction estimates from the current study are more modest than those in prior reports that focused on dementia only, underlining the importance of evaluating reductions of dementia risk factors in the context of accompanying changes in mortality.

## Supplementary Information

Below is the link to the electronic supplementary material.

Supplementary Material 1
