# Supplementary material for: The impact of modifiable risk factor reduction on future dementia burden: a microsimulation modeling study
Source: Eur J Epidemiol. 2025 Aug 23;40(9):1083–93. doi: 10.1007/s10654-025-01283-0 (PMC12537620; doi:10.1007/s10654-025-01283-0)
Supplement: Supplementary file 1 — Supplementary Material 1 [file 10654_2025_1283_MOESM1_ESM.docx]

**Supplemental material with the manuscript: The impact of modifiable risk factor reduction on future dementia burden: a microsimulation modeling study**

**European Journal of Epidemiology**

Chiara C Brück, Koen de Nijs, M Arfan Ikram, Frank J Wolters, Inge MCM de Kok

Chiara C Brück¹, Koen de Nijs¹, M Arfan Ikram², Frank J Wolters² ³, Inge MCM de Kok¹

¹ Department of Public Health, Erasmus MC University Medical Center, Rotterdam, The Netherlands

² Department of Epidemiology, Erasmus MC University Medical Center, Rotterdam, The Netherlands

³ Department of Radiology & Nuclear Medicine and Alzheimer Center, Erasmus MC University Medical Center, Rotterdam, The Netherlands

Correspondence to: Chiara C Brück, [c.bruck@erasmusmc.nl](mailto:c.bruck@erasmusmc.nl)

## A: Risk Factor Calibration

In order to add mid-life hypertension and smoking to MISCAN-Dementia, several parameters had to be calibrated to fit the assumed prevalence, dementia and mortality risk of the risk factors. For the calibration we used the genetic algorithm DEAP [1].

### Prevalence Calibration

The prevalence of the risk factors was calibrated for the respective age groups, hypertension between age 40 and 59 and smoking after the age of 65. The calibrated parameters are risk factor group sizes at birth and the calibration targets are risk factor prevalence later in life, as well as dementia incidence and mortality targets. The group sizes at birth adjust for the risk factors effect on other-cause mortality to match the risk factor prevalence later in life. Figure A1 shows the prevalence calibration output.


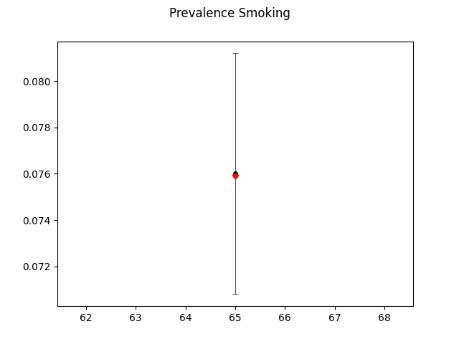

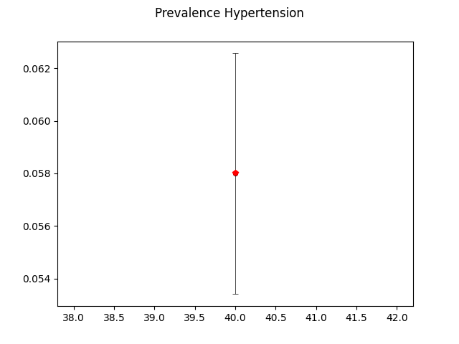

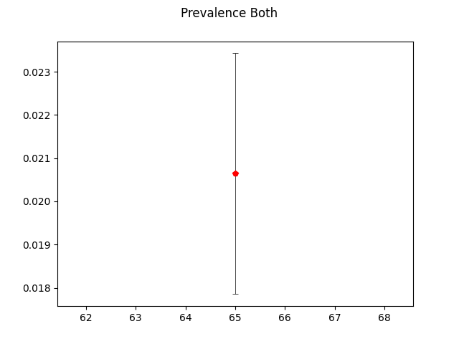


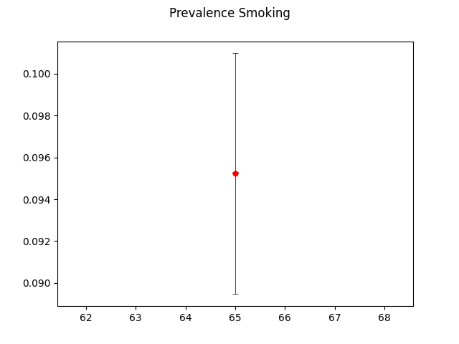

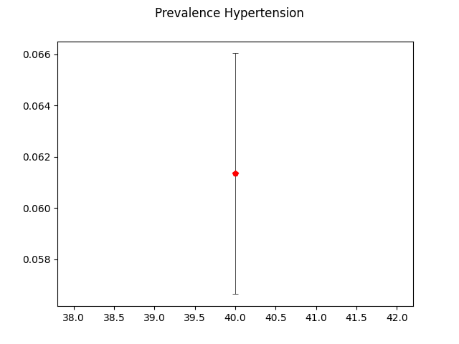

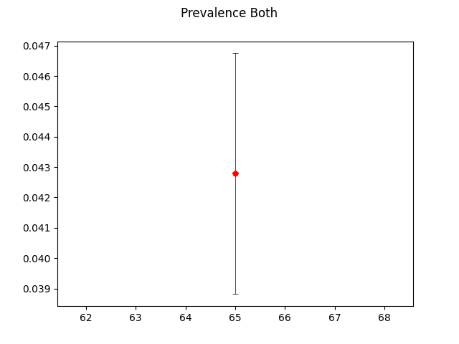


**Figure A1** Prevalence calibration output, female top and male bottom. Age-specific prevalence in model run (red dot) and prevalence rate target based on the Dutch population (black dot and confidence interval) [2-4].

### Dementia Risk Calibration

For the dementia incidence, the baseline risk for the “none” risk group was calibrated, which was then multiplied by the relative risks of the risk groups, per the literature. The overall/average dementia incidence was calibrated to equal the observed incidence rates from the Rotterdam Study [5], adjusted for a linearly declining trend as observed in a recent meta-analysis of North American and European cohort studies [6]. We assumed a linear decline in age-specific incidence of 13% per decade for the birth cohorts that contributed to the meta-analysis (1910 to 1940) and stable age-specific incidence for subsequent birth cohorts (1950 to 1980). The incorporation and calibration of this incidence trend has previously been published (referred to “scenario 3.1 nonlinear stable” in [7]). Figure A2 shows the dementia incidence calibration output for the overall population and Figure A3 shows the dementia incidence rates of the final model run.


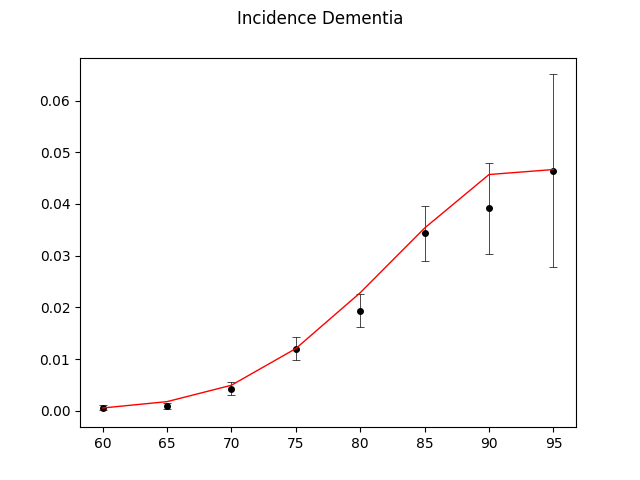

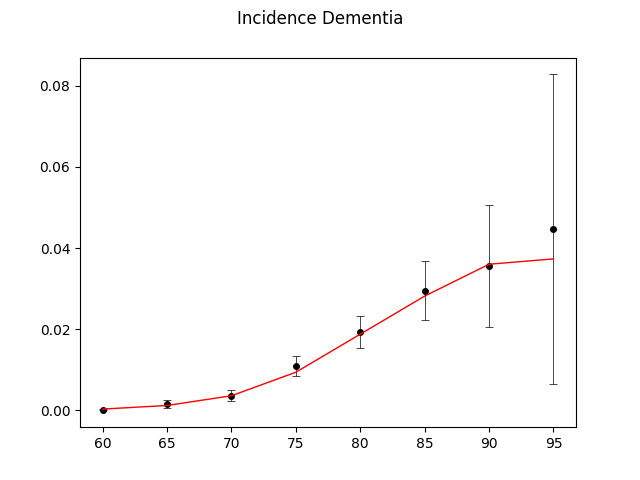


**Figure A2** Dementia risk calibration output, female left and male right. Overall dementia incidence rate by age in model run (red line) and dementia incidence rates targets based on the Rotterdam Study (black dots and confidence intervals) [5].


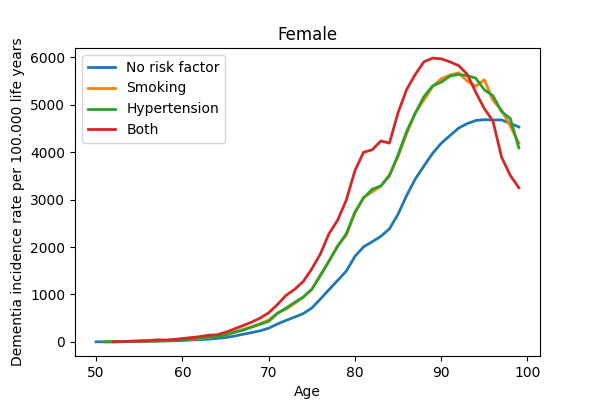

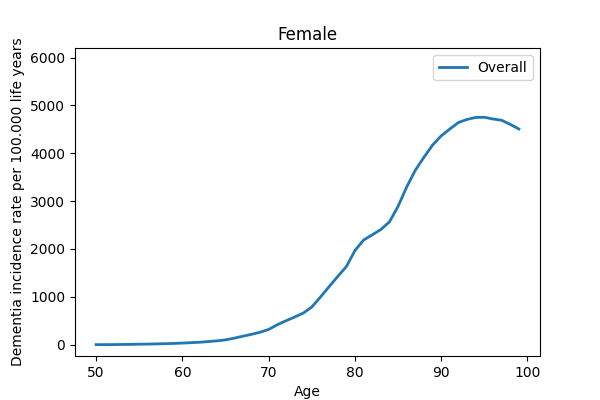

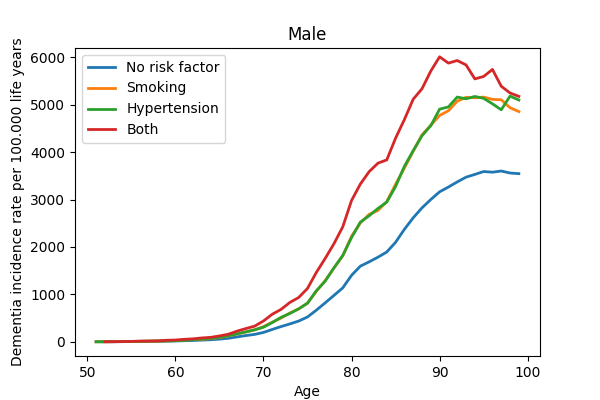

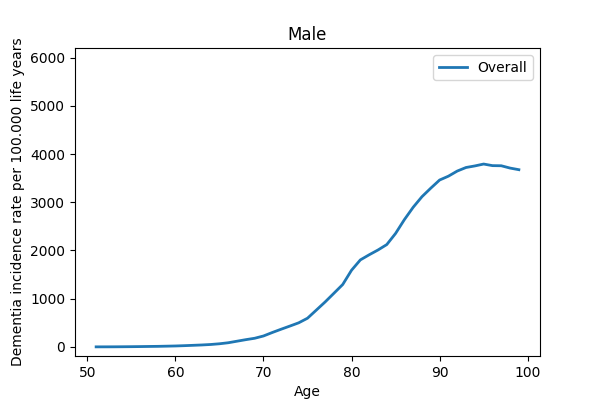


**Figure A3** Dementia incidence rates by age of the final model run, stratified by risk factor group (left)

and overall (right), female top and male bottom.

### Other Cause Mortality Calibration

All-cause mortality rates for the Dutch 1980 birth cohort by age and sex, were obtained from Statistics Netherland [8]. To account for competing risks, these mortality rates were corrected for dementia mortality through subtracting the probability of dying from dementia from the probability of dying from all causes for each age [9].

Since the risk factors that we simulate start after the age of 40, the mortality rates for all risk factor groups were equal to the overall population rates.

$$P\left( other cause mortality_{overall population},age,sex \right) = P\left( other cause mortality_{none}, age , sex \right)= P\left( other cause mortality_{smoking}, age, sex \right)= P\left( other cause mortality_{hypertension}, age, sex \right)= P\left( other cause mortality_{both}, age, sex \right)$$

From age 40 onward, the other cause mortality probabilities for the “none” risk group were assumed to be lower than those of the overall population, as the overall population includes individuals with risk factors who have higher other cause mortality probabilities compared to the “none” risk group. Therefore, the other cause mortality of the “none” group was lowered with a calibrated correction factor between 0 and 1.

$P\left( other cause mortality_{none}, age, sex \right)= P\left( other cause mortality_{overall population},age,sex \right)*Correction factor_{none}$

However, as risk factor groups have higher other cause mortality probabilities compared with “none” group, the proportion of individuals with risk factors in the overall population is expected to decrease at higher ages. Thus, at higher ages, the “none” group will represent a higher proportion of the overall population. As a result, the other cause mortality probabilities for the “none” group will converge to those of the overall population at higher ages. This convergence is assumed to start from age 70 onward and therefore, the other cause mortality probabilities of the “none” group from that age onward were assumed to be:

$P\left( other cause mortality_{none}, age, sex \right)= P\left( other cause mortality_{overall population},age,sex \right)* [Correctionfactor_{none}+\left( age-69*\left( \frac{\left( 1-Correctionfactor_{none} \right)}{30} \right) \right)]$

As indicated previously, individuals with risk factors have higher other cause mortality probabilities compared with the “none” group. Similar to the “none” group, the effect of the risk factor on mortality is expected to decrease at higher ages. As a result, the other cause mortality probabilities will converge to those of the overall population at higher ages. Thus, the other cause mortality probabilities for the risk factor groups from age 40 onward were assumed to be:

$$P\left( other cause mortality_{risk factor}, age, sex \right)=P\left( other cause mortality_{overall population}, age, sex \right) *\left( {Correction factor}_{risk factor}+[(age-40)*\left( \frac{\left( 1-{Correction factor}_{risk factor} \right)}{59} \right)] \right)$$

Where

$${Correction factor}_{hypertension} = \left\{ \begin{aligned} 1, &for age<40 \\ hypertension factor, &for age >=40 \end{aligned} \right.$$

$${Correction factor}_{smoking} = \left\{ \begin{aligned} 1, & for age<65 \\ smoking factor, & for age >=65 \end{aligned} \right.$$

$${Correction factor}_{both} = \left\{ \begin{aligned} 1, \\ hypertension factor, \\ hypertension + smoking factor, \end{aligned} \right.{for age <40 \atop\begin{aligned} for 40 <=age <65 \\ for age >=65 \end{aligned}}$$

Hence, the calibrated parameters are $Correction factor_{none}$, $hypertension factor$, and $smoking$

$factor$. The calibration targets were the mortality rates of the overall population by age, also given the incidence and prevalence targets. Figure A4 shows the calibration output and Figure A5 shows the resulting life tables used in the model runs.


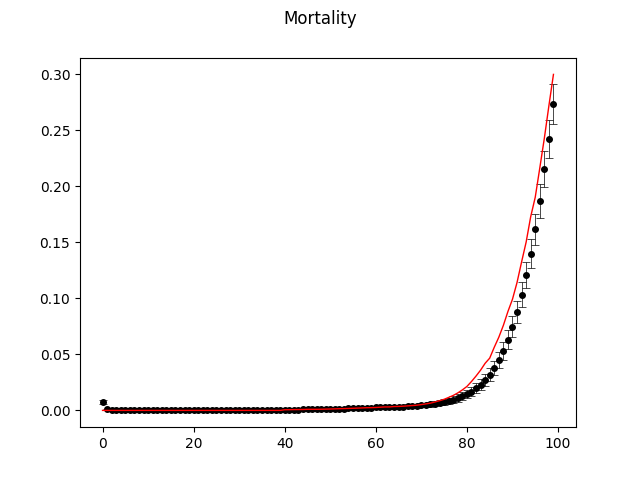

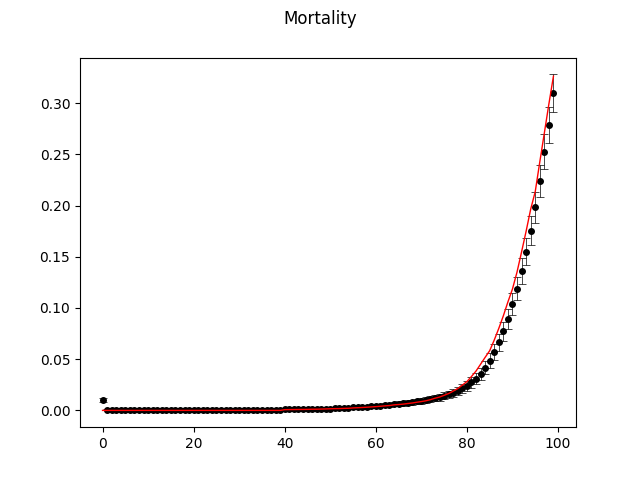


**Figure A4** Mortality calibration output, female left and male right. Overall mortality rate by age in model run (red line) and mortality rates targets representative of the Dutch population (black dots and confidence intervals) [8].


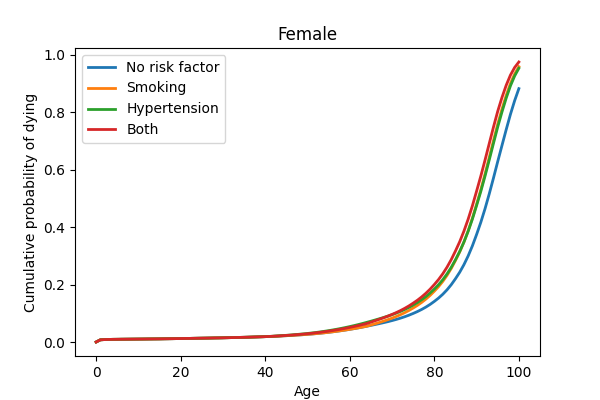

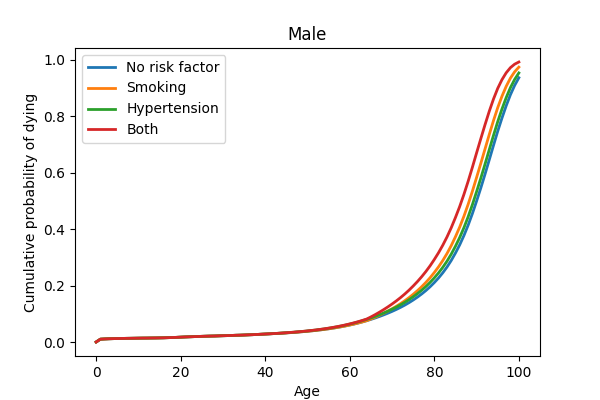


**Figure A5** Calibrated life tables by risk factor group.


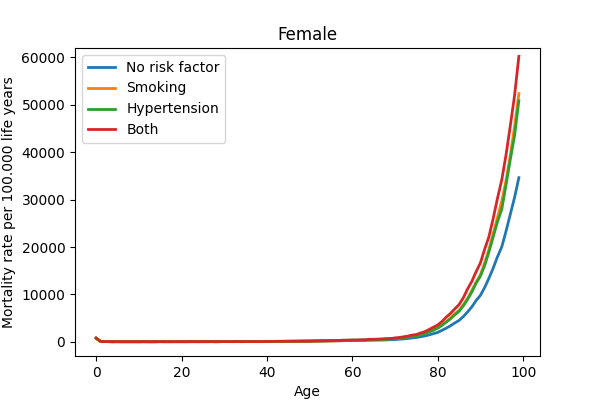

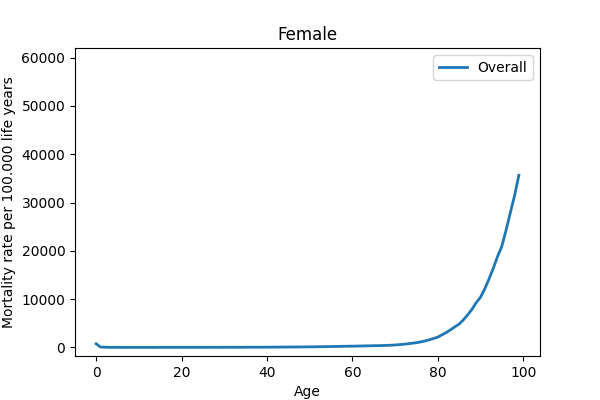

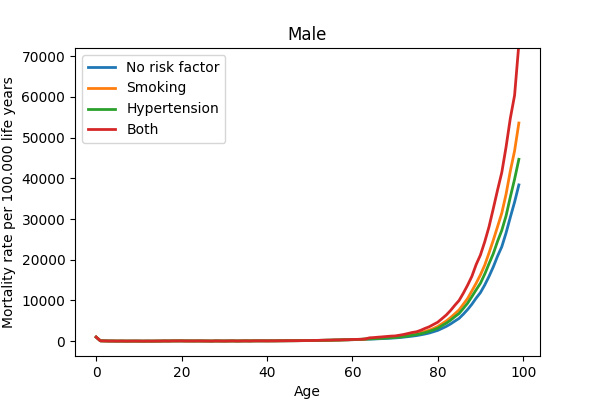

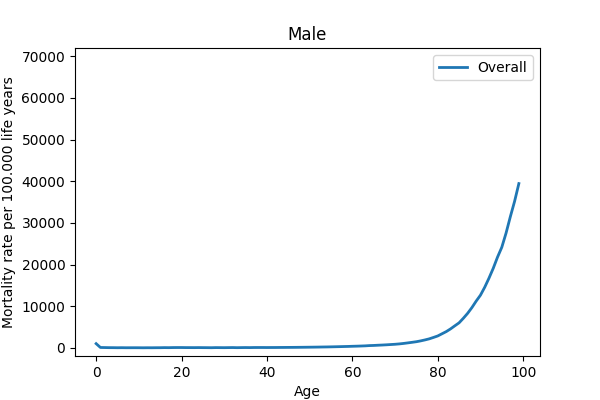


**Figure A6** Mortality rates by age of the final model run stratified by risk factor group (left) and overall (right), female top and male bottom.

## B: Sensitivity Analysis

|  |  |  | Age adjusted incidence rate | Age adjusted prevalence rate | Total dementia cases, in million | Life years with dementia,  in million | Life years without dementia,  in million |
| --- | --- | --- | --- | --- | --- | --- | --- |
| Female |  | Ref. | 0.92 | 1.54 | 21.64 | 84.19 | 8109.24 |
|  | Smoking | 10% | 0.92 (-0.33%) | 1.53 (-0.56%) | 21.64 (-0.39%) | 84.19 (-0.47%) | 8109.24 (0.01%) |
|  |  | 25% | 0.91 (-1.07%) | 1.52 (-1.17%) | 21.55 (-1.06%) | 83.80 (-1.13%) | 8109.78 (0.02%) |
|  |  | 50% | 0.90 (-2.27%) | 1.51 (-2.22%) | 21.41 (-2.13%) | 83.24 (-2.33%) | 8110.57 (0.03%) |
|  |  | 100% | 0.88 (-4.61%) | 1.48 (-4.19%) | 21.17 (-4.23%) | 82.23 (-4.61%) | 8111.92 (0.07%) |
|  | Hyper. | 10% | 0.92 (-0.31%) | 1.53 (-0.53%) | 20.72 (-0.37%) | 80.32 (-0.38%) | 8114.58 (0.01%) |
|  |  | 25% | 0.91 (-0.83%) | 1.52 (-1.01%) | 21.56 (-0.81%) | 83.88 (-0.89%) | 8110.00 (0.02%) |
|  |  | 50% | 0.90 (-1.80%) | 1.51 (-1.84%) | 21.46 (-1.66%) | 83.45 (-1.79%) | 8111.10 (0.05%) |
|  |  | 100% | 0.89 (-3.69%) | 1.49 (-3.52%) | 21.28 (-3.32%) | 82.68 (-3.58%) | 8112.94 (0.08%) |
|  | Both | 10% | 0.91 (-0.76%) | 1.53 (-0.92%) | 20.92 (-0.77%) | 81.18 (-0.82%) | 8115.88 (0.02%) |
|  |  | 25% | 0.90 (-2.04%) | 1.51 (-2.03%) | 21.47 (-1.89%) | 83.51 (-2.05%) | 8110.51 (0.04%) |
|  |  | 50% | 0.88 (-4.15%) | 1.48 (-3.86%) | 21.23 (-3.76%) | 82.47 (-4.08%) | 8112.43 (0.08%) |
|  |  | 100% | 0.84 (-8.35%) | 1.42 (-7.49%) | 20.82 (-7.50%) | 80.76 (-8.13%) | 8115.62 (0.16%) |
| Male |  | Ref. | 0.71 | 1.14 | 15.02 | 51.75 | 7952.73 |
|  | Smoking | 10% | 0.7 (-1.08%) | 1.14 (-0.4%) | 15.02 (-0.54%) | 51.75 (-0.57%) | 7952.73 (0.01%) |
|  |  | 25% | 0.69 (-2.12%) | 1.12 (-1.33%) | 14.94 (-1.45%) | 51.46 (-1.52%) | 7953.7 (0.03%) |
|  |  | 50% | 0.68 (-3.73%) | 1.11 (-2.81%) | 14.8 (-2.82%) | 50.97 (-2.91%) | 7955.17 (0.06%) |
|  |  | 100% | 0.66 (-7.02%) | 1.07 (-5.81%) | 14.6 (-5.68%) | 50.25 (-5.92%) | 7957.61 (0.12%) |
|  | Hyper. | 10% | 0.7 (-0.99%) | 1.14 (-0.34%) | 14.17 (-0.52%) | 48.69 (-0.49%) | 7962.47 (0.01%) |
|  |  | 25% | 0.7 (-1.75%) | 1.13 (-1.06%) | 14.94 (-1.23%) | 51.5 (-1.25%) | 7953.14 (0.01%) |
|  |  | 50% | 0.69 (-3.03%) | 1.11 (-2.35%) | 14.84 (-2.43%) | 51.11 (-2.5%) | 7953.77 (0.03%) |
|  |  | 100% | 0.67 (-5.61%) | 1.09 (-4.81%) | 14.66 (-4.75%) | 50.46 (-4.9%) | 7954.81 (0.06%) |
|  | Both | 10% | 0.7 (-1.61%) | 1.13 (-0.92%) | 14.31 (-1.04%) | 49.22 (-1.04%) | 7957.57 (0.02%) |
|  |  | 25% | 0.69 (-3.37%) | 1.11 (-2.57%) | 14.86 (-2.62%) | 51.22 (-2.71%) | 7954.1 (0.04%) |
|  |  | 50% | 0.67 (-6.33%) | 1.08 (-5.35%) | 14.63 (-5.28%) | 50.35 (-5.47%) | 7956.2 (0.09%) |
|  |  | 100% | 0.62 (-12.16%) | 1.02 (-10.78%) | 14.23 (-10.58%) | 48.93 (-10.95%) | 7959.68 (0.17%) |

**Table B1** Dementia outcomes by risk factor scenario compared to reference scenario, until age 85.

## C: Population sizes and life-time probabilities

| Age | Ref. | Smoking | | | | Hypertension | | | | Both | | | |
| --- | --- | --- | --- | --- | --- | --- | --- | --- | --- | --- | --- | --- | --- |
|  |  | **10%** | **25%** | **50%** | **100%** | **10%** | **25%** | **50%** | **100%** | **10%** | **25%** | **50%** | **100%** |
| 0 | 1000 | 1000 | 1000 | 1000 | 1000 | 1000 | 1000 | 1000 | 1000 | 1000 | 1000 | 1000 | 1000 |
| 50 | 973 | 973 | 973 | 973 | 973 | 973 | 973 | 973 | 973 | 973 | 973 | 973 | 973 |
| 55 | 966 | 966 | 966 | 966 | 966 | 966 | 966 | 966 | 966 | 966 | 966 | 966 | 966 |
| 60 | 955 | 955 | 955 | 955 | 955 | 955 | 955 | 956 | 956 | 955 | 955 | 956 | 956 |
| 65 | 941 | 941 | 941 | 941 | 941 | 941 | 941 | 941 | 942 | 941 | 941 | 941 | 942 |
| 70 | 922 | 922 | 922 | 922 | 923 | 922 | 923 | 923 | 924 | 922 | 923 | 923 | 925 |
| 75 | 891 | 892 | 892 | 892 | 893 | 892 | 892 | 893 | 894 | 892 | 893 | 894 | 896 |
| 80 | 832 | 832 | 833 | 834 | 836 | 832 | 833 | 834 | 836 | 833 | 834 | 836 | 841 |
| 85 | 711 | 711 | 713 | 715 | 719 | 711 | 712 | 714 | 718 | 712 | 714 | 718 | 726 |
| 90 | 504 | 505 | 507 | 510 | 516 | 505 | 506 | 509 | 514 | 506 | 509 | 515 | 526 |
| 95 | 246 | 247 | 248 | 251 | 256 | 246 | 248 | 250 | 255 | 247 | 251 | 256 | 266 |

**Table C1** Female population sizes per risk factor scenario at age 0, 50, 55, 60, 65, …, 90, 95 scaled to 1000 individuals born.

| Age | Ref. | Smoking | | | | Hypertension | | | | Both | | | |
| --- | --- | --- | --- | --- | --- | --- | --- | --- | --- | --- | --- | --- | --- |
|  |  | **10%** | **25%** | **50%** | **100%** | **10%** | **25%** | **50%** | **100%** | **10%** | **25%** | **50%** | **100%** |
| 0 | 1000 | 1000 | 1000 | 1000 | 1000 | 1000 | 1000 | 1000 | 1000 | 1000 | 1000 | 1000 | 1000 |
| 50 | 962 | 962 | 962 | 962 | 962 | 962 | 962 | 962 | 962 | 962 | 962 | 962 | 962 |
| 55 | 953 | 953 | 953 | 953 | 953 | 953 | 953 | 953 | 953 | 953 | 953 | 953 | 953 |
| 60 | 939 | 939 | 939 | 939 | 939 | 939 | 940 | 940 | 940 | 939 | 940 | 940 | 940 |
| 65 | 919 | 919 | 919 | 919 | 919 | 919 | 919 | 919 | 919 | 919 | 919 | 919 | 920 |
| 70 | 888 | 888 | 889 | 889 | 890 | 888 | 888 | 889 | 889 | 888 | 889 | 890 | 891 |
| 75 | 842 | 842 | 843 | 844 | 846 | 842 | 842 | 842 | 844 | 842 | 843 | 844 | 847 |
| 80 | 764 | 765 | 766 | 768 | 772 | 764 | 765 | 765 | 767 | 765 | 767 | 769 | 774 |
| 85 | 626 | 627 | 629 | 632 | 638 | 626 | 627 | 628 | 632 | 628 | 630 | 635 | 643 |
| 90 | 410 | 412 | 414 | 418 | 426 | 411 | 412 | 413 | 418 | 412 | 416 | 421 | 432 |
| 95 | 175 | 177 | 178 | 182 | 188 | 176 | 177 | 178 | 182 | 177 | 180 | 184 | 193 |

**Table C2** Male population sizes per risk factor scenario at age 0, 50, 55, 60, 65, …, 90, 95 scaled to 1000 individuals born.

|  | Female | | Male | |
| --- | --- | --- | --- | --- |
| Reference | 54.50% | *ref* | 35.52% | *ref* |
| Smoking 10% | 54.43% | -0.13% | 35.44% | -0.23% |
| Smoking 25% | 54.32% | -0.33% | 35.29% | -0.65% |
| Smoking 50% | 54.13% | -0.68% | 35.07% | -1.27% |
| Smoking 100% | 53.77% | -1.34% | 34.62% | -2.53% |
| Hypertension 10% | 54.43% | -0.13% | 35.39% | -0.37% |
| Hypertension 25% | 54.33% | -0.31% | 35.19% | -0.93% |
| Hypertension 50% | 54.17% | -0.61% | 34.86% | -1.86% |
| Hypertension 100% | 53.84% | -1.21% | 34.34% | -3.32% |
| Both 10% | 54.35% | -0.28% | 35.30% | -0.62% |
| Both 25% | 54.15% | -0.64% | 34.97% | -1.55% |
| Both 50% | 53.79% | -1.30% | 34.41% | -3.13% |
| Both 100% | 53.09% | -2.59% | 33.29% | -6.28% |

**Table C3** Lifelong probability of dementia for an individual free of dementia at age 65.

## References

1. Fortin F-A, De Rainville F-M, Gardner M-AG, Parizeau M, Gagné C. DEAP: Evolutionary algorithms made easy. The Journal of Machine Learning Research. 2012;13(1):2171-5.

2. Statistics Netherlands. Leefstijl; geslacht, leeftijd, persoonskenmerken [Smoking]. 2023. <https://opendata.cbs.nl/StatLine/#/CBS/nl/dataset/85464NED/table?ts=1708093924968>. Accessed April 4th, 2024.

3. Statistics Netherlands. Personen met verstrekte geneesmiddelen; leeftijd en geslacht [Hypertension Medication]. 2023. <https://opendata.cbs.nl/StatLine/#/CBS/nl/dataset/81071ned/table?ts=1708092345956>. Accessed April 4th, 2024.

4. Statistics Netherlands. Personen naar bij de huisarts bekende diagnose; leeftijd, geslacht [Hypertension]. 2023. <https://opendata.cbs.nl/StatLine/#/CBS/nl/dataset/83110NED/table?ts=1708092250843>. Accessed April 4th, 2024.

5. Ikram MA, Kieboom BCT, Brouwer WP, et al. The Rotterdam Study. Design update and major findings between 2020 and 2024. Eur J Epidemiol. 2024;39(2):183-206. <https://doi.org/10.1007/s10654-023-01094-1>

6. Wolters FJ, Chibnik LB, Waziry R, et al. Twenty-seven-year time trends in dementia incidence in Europe and the United States: The Alzheimer Cohorts Consortium. Neurology. 2020;95(5):e519-e31. <https://doi.org/10.1212/WNL.0000000000010022>

7. Brück CC, Wolters FJ, Arfan Ikram M, de Kok IM. Projected prevalence and incidence of dementia accounting for secular trends and birth cohort effects: a population-based microsimulation study. European journal of epidemiology. 2022. <https://doi.org/https://doi.org/10.1007/s10654-022-00878-1>

8. Statistics Netherlands. Levensverwachting; geslacht, leeftijd (per jaar en periode van vijf jaren). 2020. <https://opendata.cbs.nl/statline/#/CBS/nl/dataset/37360ned/table?ts=1600695663107>. Accessed December 4th, 2020.

9. Statistics Netherlands. Overledenen; doodsoorzaak (uitgebreide lijst), leeftijd, geslacht. 2020. <https://opendata.cbs.nl/statline/#/CBS/nl/dataset/7233/table?ts=1615054270240>. Accessed April 1st, 2021.
